# Exploring the use of visual predictions in social scenarios while under anticipatory threat

**DOI:** 10.1038/s41598-024-61682-3

**Published:** 2024-05-13

**Authors:** Fábio Silva, Sérgio Ribeiro, Samuel Silva, Marta I. Garrido, Sandra C. Soares

**Affiliations:** 1 William James Center for Research, Department of Education and Psychology, University of Aveiro, Universidade de Aveiro, 3810-193 Aveiro, Portugal; 2 Department of Education and Psychology, University of Aveiro, Aveiro, Portugal; 3 IEETA, DETI, University of Aveiro, Aveiro, Portugal; 4 Melbourne School of Psychological Sciences, The University of Melbourne, Melbourne, VIC Australia; 5 Graeme Clark Institute for Biomedical Engineering, The University of Melbourne, Melbourne, Australia

**Keywords:** Anxiety, Expectations, Social communication, Visual perception, Human behaviour, Emotion, Perception

## Abstract

One of the less recognized effects of anxiety lies in perception alterations caused by how one weighs both sensory evidence and contextual cues. Here, we investigated how anxiety affects our ability to use social cues to anticipate the others’ actions. We adapted a paradigm to assess expectations in social scenarios, whereby participants were asked to identify the presence of agents therein, while supported by contextual cues from another agent. Participants (N = 66) underwent this task under safe and threat-of-shock conditions. We extracted both criterion and sensitivity measures as well as gaze data. Our analysis showed that whilst the type of action had the expected effect, threat-of-shock had no effect over criterion and sensitivity. Although showing similar dwell times, gaze exploration of the contextual cue was associated with shorter fixation durations whilst participants were under threat. Our findings suggest that anxiety does not appear to influence the use of expectations in social scenarios.

## Introduction

Anxiety has been widely studied and the burden associated with it, for the individual and society itself, cannot be overstated^1^. It is no surprise then, that a vast amount of published literature is dedicated to exploring the behavioral and cognitive ramifications of pathological anxiety and high dispositional anxiety (i.e., high trait anxiety). Although not entirely aligned, the conclusions gathered from the literature for these two types of anxiety have underscored its cognitive effects, both adaptive and disruptive. One such effect concerns attention, specifically, how one is more prone to distractors as well as exhibiting greater difficulty in directing and maintaining attentional focus^2,3^. Another cognitive change typically reported as resulting from anxiety (but not always)^4^ concerns a heightened sensory-perceptual processing, showcasing a greater ability to detect (process) and perceive sudden or minor changes in our environment^5–7^.

An area of literature that remains less explored, however, concerns how normative (non-pathological/functional) anxious states affect cognition^8^. Whilst research has shown that certain cognitive changes mimic those found in individuals with anxiety disorders or high dispositional anxiety, some differences have also been found^3,9^. Nonetheless, the cognitive processes most notoriously affected by high state-anxiety remain the sensory-perceptual and attentional processes.

Attentional control seems to be improved under threat-of-shock, with studies reporting (but not always)^10^ a reduced interference from distractors^11,12^ and an increased sustained attention (enhanced vigilance)^13,14^. As for sensory-perceptual processing, this mechanism also appears to be enhanced in threat induced anxiety. This is supported by several distinct paradigms showing amplified and accelerated cortical activation during threat states^15,16^. Moreover, this is further seen in studies showing increased mismatch negativity (MMN) evoked potentials, where one’s brain response to deviant/unexpected stimuli is enhanced^17,18^. Indeed, this is even observed prior to any cortical involvement as shown by increased brainstem auditory evoked potentials^19^. Importantly, these results indicate a heightened response to stimulus novelty, supporting the idea that anxious states bolster/prioritize a sensory-driven perception. Such change will be translated into an improved ability to detect and process changes in our sensory environment. This shift towards a sensory-driven perception, however, entails (albeit not often observed)^20^ a lesser role of pre-established visual expectations over perception^17,18^. In other words, by upweighting sensory processing—hastening the detection of sensory changes—states of anxiety might limit the use of contextual cues, leading to impairments in the perception of more ambiguous and complex scenes (see below). Put differently, anxiety might provide a greater capture of sensory input at the expense of a reduced discrimination (i.e., sensitivity over specificity), since expectations based on context are less impactful during visual perception. This aligns with the idea that, in threatening situations, an increase in false-positives is an adequate price to pay to reduce any false-negatives^21,22^.

Importantly, managing a proper weighing between visual expectations (e.g., learned and present contextual cues) and sensory input is of key importance towards a quicker and more efficient visual processing^23–25^. In conditions where visual stimuli are unambiguous, or under new and unpredictable scenarios, expectations will carry less bias (subtler influence) over visual perception. However, in situations where sensory input is less reliable or complex, such as when interpreting ambiguous or noisy visual information (e.g., hearing someone talk in a loud place), expectations make a larger contribution when forming our perceptual scenery^26^.

One particularly relevant case concerns scenarios of social communication^27^. Given the natural ambiguity of social communication, where gestures and speech can have multiple meanings depending on social context, expectations are thought to have a vital role in deciphering and attributing meaning to social communication^28,29^. A study by Manera and colleagues^30^ showed this remarkably well by making participants determine the presence of a masked agent (under noise). Importantly, this agent (person) was positioned in either a communicative setting, with a second agent acting/gesturing towards the position where the to-be identified agent would (if present) be located, or with the second agent acting individually. They showed that when inserted in a communicative context (compared to an individual one), participants more often (even if erroneously) reported seeing the masked agent, depicting a reduced response criterion (*c*; more bias towards signaling presence).

Thus, although a more sensory-driven processing during anxiety (downplaying expectations) might be advantageous in certain situations, it remains unclear how these changes (i.e., a more sensory-driven perception) might affect the individual in social circumstances. Here we specifically raise the question of how (and if) our ability to interpret ambiguous social actions is compromised when under anxiety. We plan on answering this question by comparing how individuals under threat of shock (an anxiety inducing condition), compared with safe conditions, extract and use cues from social gestures to infer the presence of a second agent (under noise) that might be partaking in a social interaction with a first agent (similar to the task described above)^30^. We will rely on two signal detection theory measurements^31^: criterion (*c*), which captures one’s response bias, and here reliance upon social gestures (context)^30^; sensitivity (*d*’) which reflects one’s ability to discriminate between signal and noise, and provides a proxy over heightened sensory-perceptual processes^32^. Based on the literature above we expect that, under threat induced anxiety, participants will be less reliant on (less influenced by) the actions of a communicative agent compared to safe conditions. Specifically, we posit that (1) in safe contexts/blocks (but not during threat blocks), criterion will be lower (more bias towards signaling presence) in communicative actions compared to individual scenarios, and (2) in communicative actions, the criterion will be lower in the safe compared to the threat contexts. Furthermore, as an exploratory measurement, we employed the use of an eye tracker to investigate the visual exploration patterns in these social scenes and comparing them across threat and safe conditions.

## Methods

### Participants

The sample size used in this study was estimated based on 2000 simulations assuming expected means and standard deviations (estimated with the aid of prior studies)^33^. To achieve this, we used the Superpower package for R (0.2.0)^34^. Given these parameters, and assuming our statistical design (2 by 2 within-subjects) and desired power of 0.8 (partial eta-square of 0.15), this method yielded a minimum required sample size of 65 participants.

To be included, participants needed to be between 18 and 40 years of age, Portuguese speakers, and having normal (or corrected) eyesight. They also needed to have no past or current diagnosis of any neurologic or psychiatric disorders, and not to be taking any relevant medication (e.g., for anxiety or depression). A total of 72 participants were initially recruited for the experimental session. All participants were recruited via institutional e-mails and social media. Of these, one was excluded for disclosing taking anti-depressive medication after the study’s conclusion, four for reporting the presence of agent B in less than 5% of the trials (where agent B was actually present) and one participant for showing a low identification accuracy (i.e., less than our predefined threshold of 75%) regarding the type of actions (communicative vs individual). Our final sample consisted of 66 participants (52 female; M_age_ = 21, SD_age_ = 2.3). The present study was conducted with permission from the ethics committee (reference 18-CED/2020) and in accordance with the data protection regulation from the University of Aveiro.

### Stimuli and apparatus

The experimental task was presented on an MSI Pro MP241 monitor with a 1920 by 1080 pixels resolution and programmed using Psychopy version 2021.2.3^35^. Behavioral responses were given through a standard QWERTY keyboard. The data from online questionnaires were collected using the Limesurvey platform. The eye tracker used was a Gazepoint GP3 (150 Hz).

The device used for current stimulation (electric shock delivery) was a Biopac STMISOLA module. The electric shocks were administered via two electrode pads placed in the participants’ forearms. The electric shock intensity was controlled, ranging from 2 to 6 mA, with a 100 ms duration for each stimulation.

For the communicative and individual actions we used videos of point-light displays of human figures from the Communicative Interaction Database^36^. Each person (agent) was represented by 13 white dots attached to the major joints and head. Prior to usage in the main experimental task, we performed a brief pilot study^36^ for the Portuguese population (N = 31) that allowed us to select the actions that were, on average, most accurately discriminated (less error-prone). We selected “drink”, “lateral steps” and “turnover” for our sample of individual actions, and “sit down”, “pick this up” and “squat down” for the communicative actions sample. Subsequently, the dots for the selected actions were manipulated using Matlab (R2019b) to create versions with one of the agents (agent B; see below) under a noise mask that varied in the number of dots. Similar to Manera^30^, a limited-lifetime technique was also implemented, making a maximum of six randomly chosen dots of the agent visible, at any time, for 200 ms; dot appearance and disappearance were asynchronous across dots. Additional versions of these videos were created, where the dots representing agent B were also temporally and spatially scrambled while keeping their trajectory and velocity, effectively removing a coherent representation of the agent from the scene (absence condition).

The noise masks consisted of a fixed number of dots randomly added over the agent (the number of noise dots was adjusted to each participant; see below) and adopted a limited-lifetime technique. Each of the noise dots was built by sampling a random 200 ms interval of an agent’s dot trajectory, over the complete action of the agent, and placing it in a random position. This meant that each noise dot was present for 200 ms, being replaced by another noise dot, afterwards, and had a trajectory and velocity akin to the agent’s dots. To further prevent any familiarity due to stimulus repetition, each type of action had five versions, each exhibiting different limited-lifetime agent sequences and noise masks. Each video was presented occupying 12.5 by 18 visual degrees and averaging 4.3 s in length. This same video creation process was performed for the set of three videos used in the adjustment phase (with only one agent present, this time).

### Procedure

Prior to their lab session, participants filled out a brief online form, providing their informed consent and completing socio-demographic information (i.e., age, sex, currently diagnosed diseases, etc.), the trait part of the State-Trait Inventory for Cognitive and Somatic Anxiety (STICSA)^37,38^ and the Liebowitz Social Anxiety Scale (LSAS)^39,40^. If eligible, they were then contacted, and the experimental session was scheduled.

In the experimental session participants began by completing the STICSA-State, followed by the shock calibration procedure. Here, participants received a graded series of electric shocks, starting at 2 mA and going up to a maximum of 6 mA. After each shock, participants were asked to indicate how uncomfortable that shock felt on a visual paper scale (from 1 “barely felt” to 5 “very unpleasant/uncomfortable”). The shock intensity was increased in steps of 1 mA until they reported a rating of four (“quite unpleasant/uncomfortable”) or the 6 mA (maximum) level was reached. If the rating of four was reported before the 6 mA level, shocks of that same intensity were administered until five shocks in total (since the beginning of the workup procedure) had been delivered. The intensity for the electric shock defined in this task was kept constant throughout the main task.

Participants were positioned in front of the computer screen and asked to position their heads on the chinrest, adjusting the chair, if needed. A brief eye tracker calibration was then performed. Two more calibrations were also done, one just prior and one in the middle of main task (roughly ten minutes apart).

Participants were then introduced to the main computerized task. The task began with an adjustment phase (60 trials), where the amount of noise dots each participant would be exposed to during the experimental task was determined. Participants were presented with a video of a point-light display of either an agent performing a simple individual action (an agent looking under their foot, sneezing or stretching) or a scrambled agent (absent condition), both superimposed by five different noise mask levels in total (i.e., 5, 15, 25, 35 or 45 noise dots). These actions were only used during this phase. Thus, the agent could either be present (agent plus noise dots) or absent (scrambled agent plus noise dots). After watching each video (average duration of five seconds), participants were tasked with indicating if any agent was present using the mouse cursor (no time limit). The level selected, which would be used throughout the experiment, was the noise level that reached an accuracy level closest to 75%.

Before starting the main experiment, participants were provided with a detailed description of the instructions, as well as some animated examples of the type of stimuli they would be exposed to during this task. In the experimental task, participants were shown videos depicting either one or two agents (see Table 1). On the left side of the video, Agent A was always present and shown clearly (i.e., without any noise or limited-lifetime technique). This agent could either perform an individual action (e.g., drinking a glass of water) or a communicative action (e.g., asking the agent beside to “look over there”). The agent to the right, Agent B could either be present or absent from the video. If present, Agent B would be shown with a limited-lifetime technique and superimposed noise mask, making their identification difficult. Furthermore, their action would either be individual, if agent A’s action was also individual, or a communicative response to agent A’s action, if Agent A was performing a communicative gesture. On the other hand, if agent B was absent, the agents’ dots would instead be spatially and temporally scrambled, and once more shown with the limited-lifetime technique and under a cloud of noise dots.

**Table 1:** Summary of the multiple conditions participants were exposed to. All trials had Agent A present, with the type of action being displayed by this agent being either communicative or individual. As for agent B, this agent could be present in the video (with noise) or absent (scrambled agent plus noise). If present, Agent B would always be performing the same type of action (communicative or individual) as agent A.

Agent A’s action	Agent B	
	Present (Agent B + Noise)	Absent (Scrambled Agent B + Noise)
Communicative	Both agents are shown performing communicative actions towards one another	Only agent A is shown and is presenting a communicative action
Individual	Both agents shown performing individual action (not involving each other)	Only agent A is shown and is presenting an individual action

After each video, a response screen was shown, and participants were tasked with indicating, using the mouse cursor, if only one (just agent A) or two agents (A and B) were present. Importantly, participants were previously told that agent A’s actions were semantically related to Agent B (if present) and that they should, therefore, direct their attention to it at the beginning of each trial. To emphasize their initial evaluation of agent A’s actions, the fixation cross presented in each inter-trial period (one second duration) was located over the position agent A would appear in. Additionally, in around 8% of the trials (two per block, see below), an additional response screen was shown, prompting the participant to answer if agent A’s action was communicative or individual in nature.

**Figure 1:**
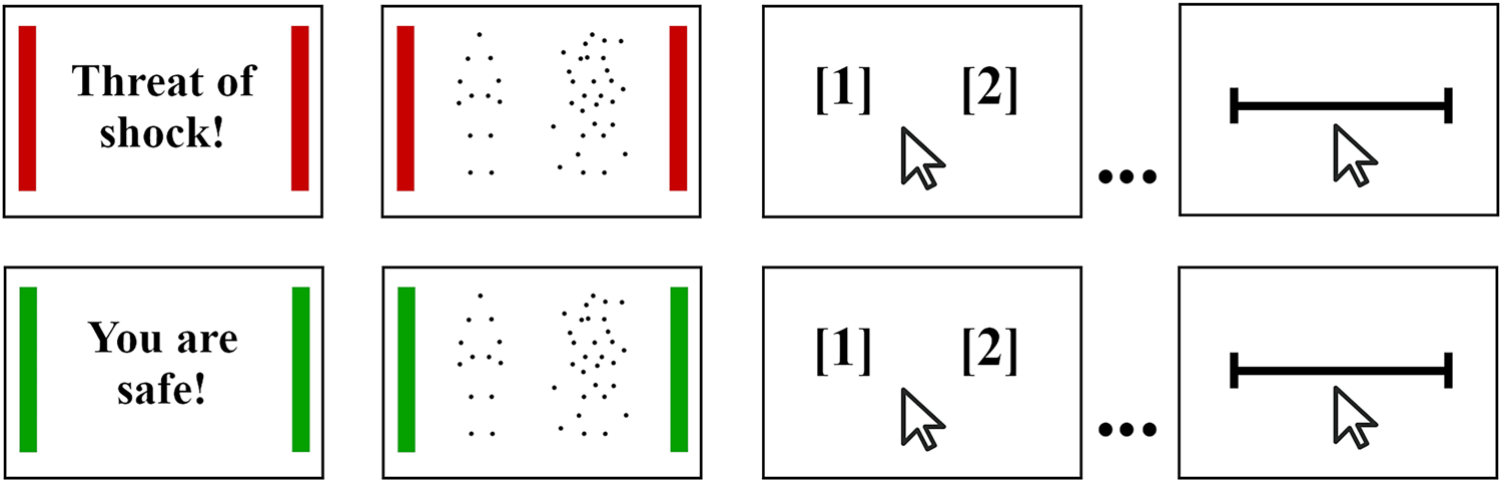
Illustrative temporal structure of a block and trial. Each block began with a warning screen, either indicating that participants would be safe during the next block or that they would be at risk of getting an electric shock. Each trial was composed of a video of one or two agents, followed by a selection screen, where participants had to decide if one or two agents were present in the video they just watched. At the end of each block they had to answer how anxious they felt during the previous block on a visual analog scale.

Lastly, it is important to note that participants completed this task under two conditions (blocks). In one type of the blocks, labeled threat blocks, participants were randomly exposed to electric shocks. This would occur in approximately 8% of the block’s trials (i.e., two trials per block); participants were unaware of how many shocks they would be receiving. The safe blocks were performed without the delivery of any electric shock. Participants were always alerted to the type of block they would be going into and, as such, were fully aware if they were at risk of receiving an electric shock or not. Furthermore, during threat and safe blocks, two lateral red and green bars, respectively, were presented, to remind participants of the block they were currently in. Six blocks (three threat and three safe blocks) with a total of 144 trials (24 per block) were presented (see Fig. 1). Blocks were presented in an alternating fashion, with the starting block (threat vs safe) being counterbalanced across participants. At the middle mark of the task, participants were encouraged to take a small break. At the end of each block, they were asked to indicate how anxious they felt on a visual analogue scale (from 0 “Not anxious” to 100 “Really anxious”).

Upon finishing the task, participants were asked to indicate, on a visual analogue scale (0–100): (1) how much, during the whole task, did they rely on agent A to gather clues as to the presence of agent B, (2) how much did they thought the actions of agent A related to the actions of agent B. Lastly, they viewed each previously seen video during the main experimental task without any video alterations (i.e., no scrambling, noise dots or limited-lifetime technique). In a final task participants watched each video/action used in the main task (without any noise) and were asked to determine the type of action in each video (communicative or individual) and choose which description, based on 5 alternatives, better described the actions in the videos adapted from 36. This was done to assess both ability to discriminate the communicative intention of the actions as well as to understand how well they were able to understand the actions.

### Statistical analysis

All behavioral data treatment and statistical analysis were performed with R (2022.02.1) and with JASP (0.16.3). From the response data as well as the type of trial (agent B present vs absent), signal detection theory measures of sensitivity and criterion were computed (psycho package)^41^. A *p*-value below 0.05 was set for statistically significant effects.

Criterion (*c*) and sensitivity (*d*′) were analyzed in two repeated-measures ANOVAs, with block and action type as fixed factors. To control/account for individual anxiety factors, we also considered the addition of STICSA (state and trait) and LSAS as covariates. The final model (judged by the significant values and AIC/BIC indices) did not include any covariates. As a measure of effect size, we used the omega squared. Residual analysis of the model was evaluated graphically and did not display any major violations from normality. For the manipulation check analysis, we performed a pairwise t-test between average anxiety ratings in the threat and safe blocks. Correlations between the anxiety inventories and the overall criterion and sensitivity were additionally explored in a separate analysis with Pearson correlations (Bonferroni corrections were used on *p*-values). Lastly, an exploration of measures gathered in the final questions regarding the usage of agent A’s cues, and how these related to agent B, are detailed in Appendix A. To assess the support for the null hypothesis, additional Bayesian analyses for the criterion and sensitivity, with the respective bayes factors for each parameter, were conducted and are provided in full in Appendix B.

Eye tracker data was extracted and processed in R. Trials where track loss was superior to 25% were removed (220 trials across all participants). Since no participant revealed an overall track loss superior to 20%, no participant was removed from the data set. Only three participants were removed due to recording issues. The final number of participants in the eye tracking data was 63. Data transformation for window time and sequential analyses was performed with the package *eyetrackingR* (0.2.0)^42^ for R. For the proportion analysis, the data were binned into 200 ms intervals and analyzed in a linear mixed model. This model had block and time bin (centered) as fixed factors, with ID as random intercepts, with respective slopes per block and time.

For the analysis of fixations, we used linear mixed models for the duration analysis and a simple paired t-test for the average count. The minimum fixation duration was established at 50ms^43^. Considering the distribution of time spent gazing at agent A, the time of interest for this analysis was limited to the first two seconds (which corresponded to ~ 80% of the time spent on agent A overall).

This study was pre-registered prior to any data collection (osf.io/nbszc). All data analyses concerning our hypothesis (criterion measure) were pre-registered analyses, with the remaining analyses (sensitivity and eye tracking data) being interpreted as exploratory analyses. All data and analysis scripts can be found in the following online repository: osf.io/6vawb.

### Ethical approval

This study was conducted with permission from the ethics committee (reference 18-CED/2020) and in accordance with the data protection regulation from the University of Aveiro.

## Results

### Behavioral and subjective results

Concerning our manipulation check, we were able to observe that participants’ subjective anxiety ratings were, on average, higher for anxiety blocks (M = 38.5, SD = 26.8), compared to safe blocks (M = 7.96, SD = 10.9; *t*(65) = − 10.18, *p* < 0.001, *d* = − 1.25, 95% CI [− 1.57, − 0.93]).

**Figure 2:**
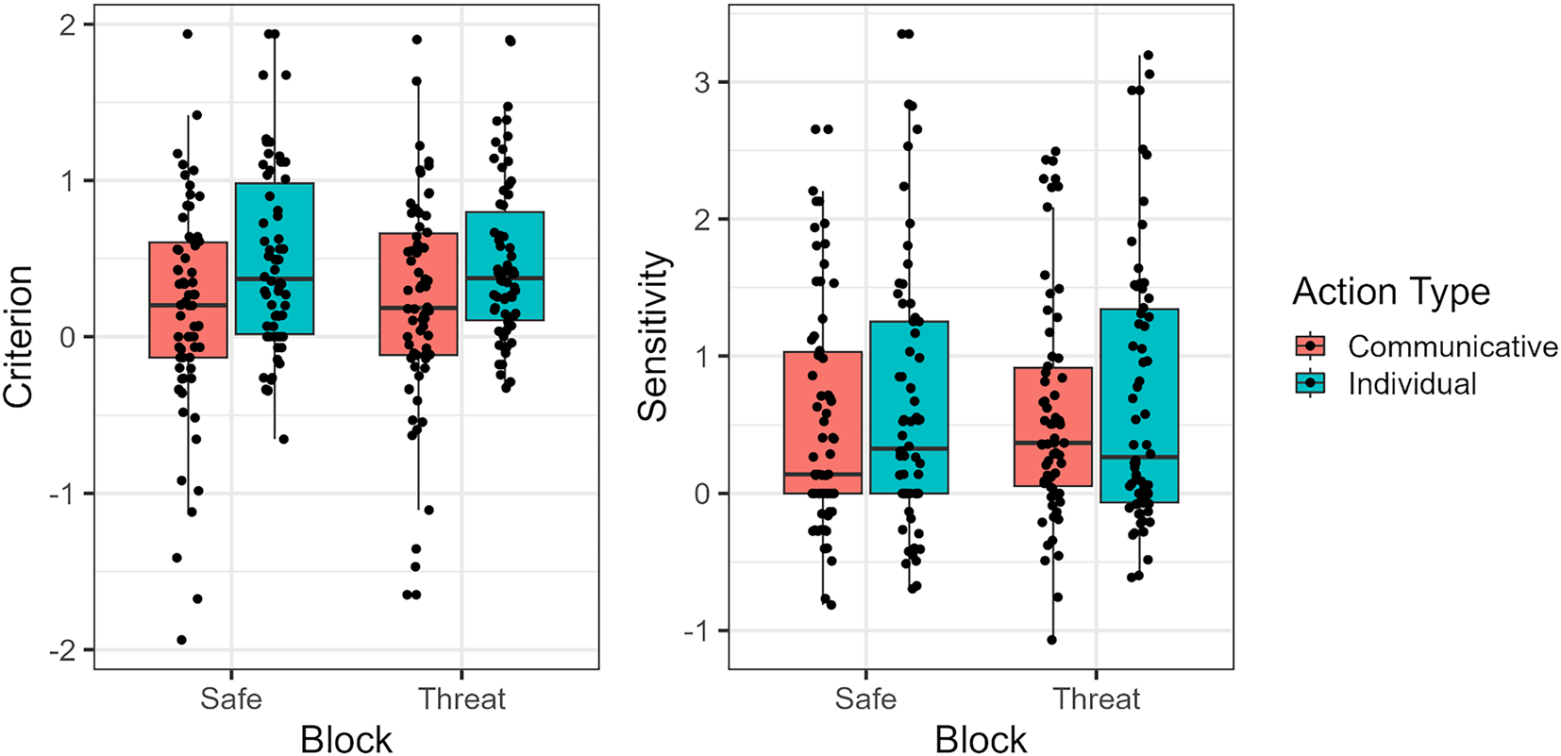
Observed criterion and sensitivity data across block and action types.

Beginning with criterion, our analysis showed that only action type had a significant effect (F(1, 65) = 9.823, *p* = 0.003, ω^2^ = 0.052, 95% CI [0.0, 0.19]; see Fig. 2), with communicative actions depicting a lower criterion than individual actions. No statistically significant effect of threat of shock (F(1, 65) = 0.270, *p* = 0.605, ω^2^ = 0, 95% CI [0.0, 0.0]), nor any interaction between this and action type (F(1, 65) = 1.425, *p* = 0.237, ω^2^ = 0, 95% CI [0.0, 0.04]), was found. This lack of effect was further supported by a Bayesian analysis with the following bayes factors: BF_01_ = 6.25 and BF_01_ = 4.9, respectively (see Appendix B for the full analysis).

Concerning sensitivity, our analysis showed that threat of shock was not significantly different from safe conditions (F(1, 65) = 0.837, *p* = 0.364, ω^2^ = 0, 95% CI [0.0, 0.0]). Action type did show a marked tendency, with increased sensitivity in individual actions, but remained not statistically significant (F(1, 65) = 3.856, *p* = 0.054, ω^2^ = 0.004, 95% CI [0.0, 0.08]). The interaction between threat of shock (vs safe) and action type was also not statistically significant (F(1, 65) = 0.054, *p* = 0.817, ω^2^ = 0, 95% CI [0.0, 0.0]; see Fig. 2). Again, this lack of effect of block was further supported by the Bayesian analysis (BF_01_ = 4.425 and BF_01_ = 3.718, respectively).

Lastly, we showed no significant correlations between state (t(64) = 0.322, *p* > 0.999), trait (t(64) =  − 1.503, *p* = 0.827) and social anxiety (t(64) = 0.482, *p* > 0.999) with criterion. In addition, no significant correlations were also found between state (t(64) = 0.267, *p* > 0.999), trait (t(64) = 0.418, *p* > 0.999) and social anxiety (t(64) =  − 1.603, *p* = 0.682) with sensitivity.

### Eye tracker

**Figure 3:**
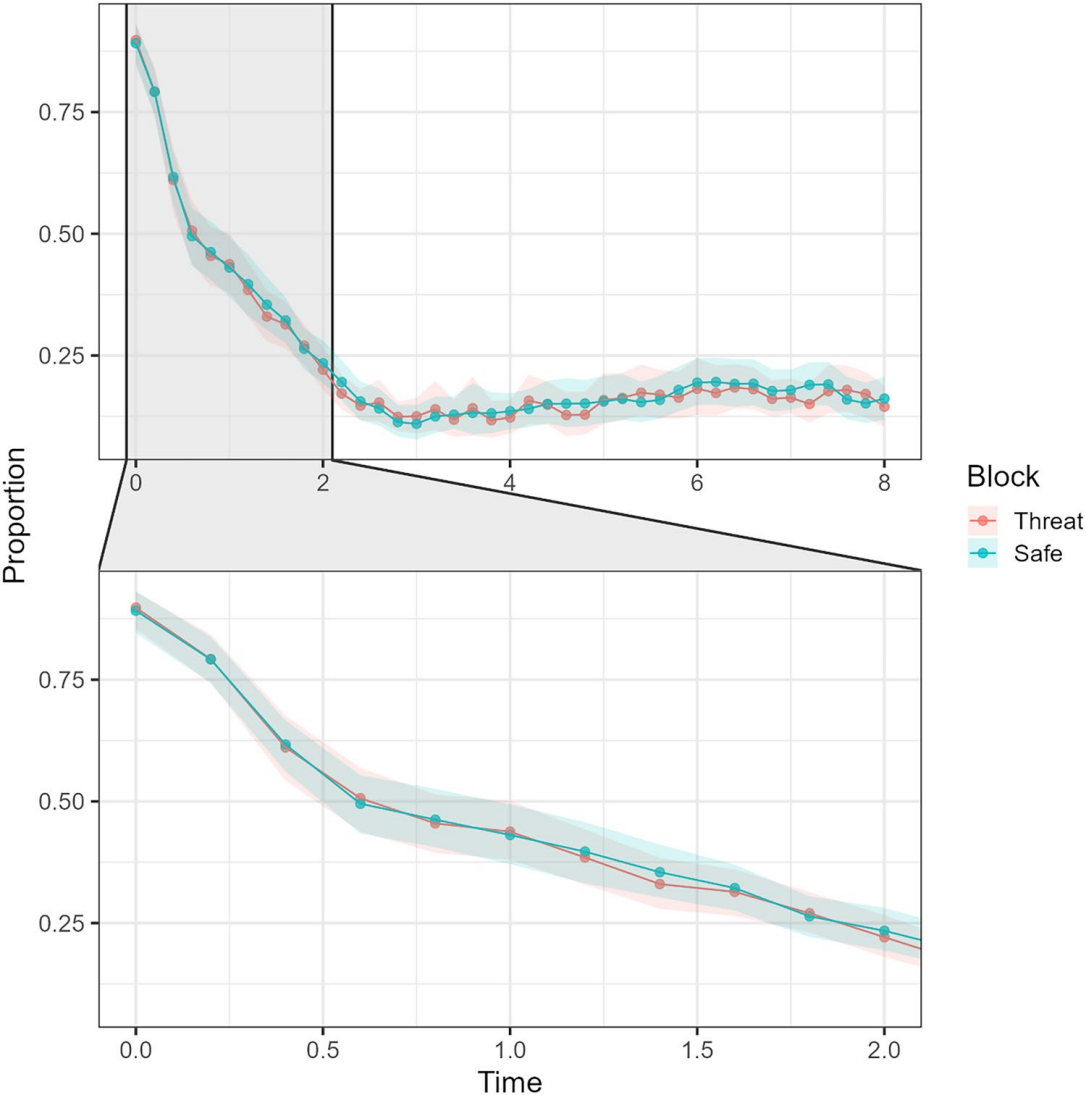
Proportion of time spent looking at agent A compared to agent B. Top image shows the maximum possible time of a trial. Bottom image shows just the first two seconds. Each point is a time-bin of 200 ms. Higher values reflect more time spent gazing at agent A.

In the first two second window of each trial, the proportion of time looking at the regions of interest containing agent A (compared to agent B) was different across time (χ2(1) = 630.69, *p* < 0.001), as expected. However, threat of shock showed no effect (χ2(1) = 0.077, *p* = 0.782), nor was there any significant interaction between this and time (χ2(1) = 0.521, *p* = 0.471; see Fig. 3).

**Figure 4:**
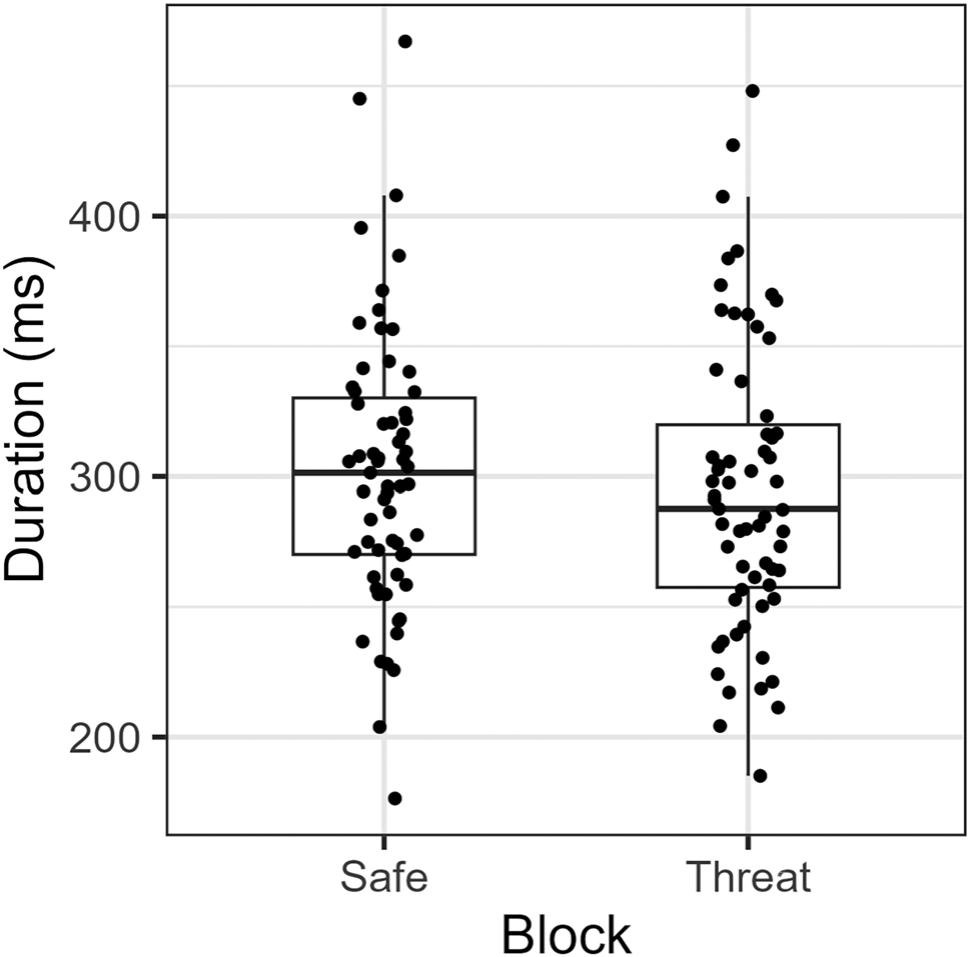
Average fixation durations (in ms) for the first two seconds for each block (safe vs. threat).

When looking at the fixations for the first two seconds of each trial, we found that the average duration of fixations on agent A were smaller during threat blocks, compared to safe blocks (χ2(1) = 4.305, *p* = 0.038; see Fig. 4). As for the number of fixations on agent A during this time period, no significant difference between blocks was found (t(62) =  − 1.248, *p* = 0.217, *d* = − 0.16, 95% CI [− 0.41, 0.09]). No statistically significant difference between threat and safe blocks in the average fixation duration (χ2(1) = 2.633, *p* = 0.105) or the number of fixations (t(62) = 1.68, *p* = 0.099, *d* = − *0.21*, 95% CI [− 0.46, 0.04]) was found for agent B after the first two seconds (two to eight seconds).

## Discussion

In this study, we investigated the extent to which anxious states affect the weight given to expectations, when interpreting the presence of a social agent in a noisy environment. To this end, we presented participants with a task where they had to judge the presence of a masked agent (B) considering the different cues (communicative vs individual) provided by a second agent (A). Importantly, this task was performed in both safe and threat-of-shock conditions. We measured the participants’ criterion and sensitivity in perceiving agent B as a function of the type of cue and condition that they were in, and we also gathered eye tracking information.

As with prior studies using this interpersonal predictive coding paradigm^30,44^, we observed an effect of action, where communicative actions displayed by agent A led to significantly lower criterion in signaling the presence of agent B. Contrary to what we expected, however, threat of shock played no significant part in shaping participants criterion (hypothesis 1). In other words, threat of shock neither affected the overall criterion nor did it moderate the effect of type of action over the criterion. Likewise, a direct comparison of the criterion shown during communicative actions across blocks (safe and threat-of-shock) revealed no significant difference between these two contexts (hypothesis 2). Overall, the results above seem to suggest that being under threat induced anxiety is not enough to affect the weight given to expectations in these types of social settings.

Albeit surprising, given prior results that suggest a more stimulus-driven processing^17,18^, we discuss possible explanations. One such explanation falls upon the differences between the measures used to capture the weight given to expectations. Whilst noticeable in terms of brain activation (evoked-related potentials) following a mismatching stimulus (i.e., in MMN responses), changes directed at the valorization of sensory input (and consequently expectations) might be harder to capture with behavioral paradigms, where subjective answers are used as markers. This is further supported by at least one other study^45^ which saw no difference in this same paradigm between schizophrenic and control patients. This was observed despite evidence of a reduced mismatch negativity associated with this disorder (schizophrenia) from other MMN-base studies^46^ and other neuroimaging studies^47^. Together with the high variation of the criterion shown by participants in our task, it might be the case that changes in expectation reliance might be harder to capture using self-reporting measures (as in our study).

In line with the above, the modality of presentation is also worthy of discussion. Most studies investigating evoked potentials to deviants (i.e., MMN) do so in the auditory domain. Visual mismatch negativity (vMMN) is considerably less explored than its acoustic counterpart^48,49^ but, nonetheless, both versions of MMN appear to share similar characteristics between them^50^. Only one study related vMMN (with emotional faces) and anxiety, investigating this relationship in patients with panic disorder^51^ and showing instead a reduced vMMN in this population. This conclusion is supported^52^ but also contradicted^53^ by other studies that use auditory MMN. Thus, it is currently hard to say if conclusions regarding MMN in the auditory modality are transposable, as expected, to paradigms using visual stimuli (as with our study).

It is also worth mentioning that, despite the majority of the studies aligning with those found by Cornwell and colleagues^17,18^, other studies exhibited contrasting results^51,52,54^. As highlighted by Fucci and colleagues^20^, this could be due to these effects manifesting themselves only on highly anxious individuals, be it in individuals with trait anxiety or in those experiencing really high levels of anxiety, who would thus show a measurable increased in sensory-driven perception. In other words, only during truly elevated states of anxiety, which might not have been fully achieved with our manipulation, or with people with elevated proneness towards experiencing anxiety, would this effect manifest itself. While in our study both state and trait anxiety did not exert a significant impact on our results (directly on the decision measurements, or over our predictors), it is possible that the overall anxiety experienced by participants (compared to other highly stressful daily situations) was still low. As such, to address this potential limitation, we recommend that future studies tackle this question by considering groups of individuals with high-trait anxiety, while also employing more objective methods to ensure elevated states of anxiety.

Other factors related to the characteristics and design of our experimental task might also have contributed to the lack of differences shown between safe and threat contexts. One such factor might pertain to the low accuracy observed. Accuracy across blocks and types of action ranged from 57 to 61%. This is a little less than intended since the calibration performed at the beginning of the task aimed at approximating accuracies towards the 75% value. One could argue that perhaps the task difficulty was higher than expected, with participants having a harder time identifying agent B. This could have led to responses, in the case of some participants, being given at random. The accuracy reported here was nonetheless similar to another study using this paradigm, in which no differences in the task between people diagnosed with schizophrenia and healthy subjects were found^45^.

In line with the low accuracy during the task, another possibility might be the arguably low accuracy observed in the attention check task (~ 77%), where participants, on random trials, had to indicate the type of action displayed by agent A. This task was meant to ensure that participants paid sufficient attention to agent A at the beginning of the trial, which might not have been to the extent desired. This is, in part, also supported by eye tracking data, which showed that in 25% of all trials (across participants) participants had less than half a second of time spent on agent A (see Appendix C). Nonetheless, we believe that this low accuracy was more a result of forgetfulness rather than of lack of attention, and that participants did, indeed, pay sufficient attention to agent A. This is supported by both the participants’ self-reports at the end of the task and, pivotal to this argument, the fact that sufficient attention had to be given to agent A for the effects of action to emerge. Thus, we see little reason to believe that the lack of attention towards agent A might be explaining the results found here.

Regarding sensitivity, we did not observe any effects from block, only showing that individual actions were marginally associated with increased sensitivity. Other studies have managed to show an opposite pattern, i.e., more sensitivity in communicative actions^33,44^, but some, as with this one, showed no significant difference^30^. Since exploring why this is the case remains outside the scope of this study, we merely highlight the need for future studies why such differences might emerge.

To rule out any effect of the self-reported anxiety on both measures discussed above (criterion and sensitivity), we also explored and showed that neither measure was associated with state, trait, or social anxiety. While past studies have hinted at a positive relation between either trait and/or state anxiety and MMN^20,55,56^, which would possibly be expected to reflect on criterion, others revealed opposite patterns^4,52^. Furthermore, many of the latter findings connecting levels of anxiety with increased or decreased amplitudes of MMN are not generally observed, but are instead dependent on the type of population (e.g., panic disorder patients) and emotional characteristics of the stimuli (e.g., fearful)^52,55,56^. Perhaps only in more severe cases (e.g., pathological population) or with negative-valence stimuli (e.g., fearful/threatening interactions) could any of the above results be reflected in terms of actual response criterion.

Concerning our gaze analysis, no apparent difference between safe and threat of shock conditions in the proportion of dwell time over agent A compared to agent B (during the first two seconds) was found. Aligned with the behavioral results, this supports the idea that the time allocated to agent A during the start of each interaction was consistent, irrespective of context. However, when exploring the average fixation duration towards agent A during the initial moments of each video, participants under threat depicted shorter fixation times than when under safe conditions. This finding aligns with literature depicting gaze behavior in different types of sports tasks, revealing that in highly anxious individuals (trait and state anxiety), the average fixation duration tends to be smaller, when compared to controls^57,58^. It is also known that, under threat, participants tend to have higher volatility regarding the fixations behavior^59^. Alongside the findings above, and the ones in this study showing a reduced fixation duration, this might suggest an increased difficulty (decrease in efficiency) in extracting information from the environment. However, this finding remains speculative, but should be considered in future studies.

Some limitations should be pointed out. One limitation pertains to the lack of a confidence rating measure regarding the participants’ response on each trial. This could have provided valuable cues as to whether responses were given at chance or to the degree of confidence deposited in each decision. It could also potentially reveal differences in response confidence between threat and safe blocks. In line with this limitation, but disregarding fatigue factors, we believe that adding more trials to the calibration phase would better fine tune the task towards each participant. Since either a too easy or too hard a task could either prevent biases from agent A from emerging or give way to random responses, respectively, this might be something to be considered in future experiments.

Another limitation worth mentioning concerns a possible lack of statistical power. This could have arisen due to two factors. One is that our power analysis might have been designed using overly optimistic estimates, which could mean that, if present, the effect we sought here is actually smaller than expected. A second factor is the limited sample of actions representing the communicative and individual actions (three per category). This is due to the limited size of available databases and to the validation of these same actions for the Portuguese population. Although we note the number of stimuli is similar to prior studies with this paradigm^44,45^, future studies should still expand existing databases and broaden the representation of their study variables.

Finally, changing the social scene’s valence, such as using actions/gestures with a positive and negative connotation, as well as using specifically threat-related actions^60^, might also be a valuable avenue for exploration. Indeed, as shown by Zheng and colleagues^54^, stimuli evoking threat appear to be a critical factor in determining increased MMN. One could hypothesize that embedding the possibility of threat within the social interaction, be it simple aggressive gestures by one of the agents or associating the probability of shock to the presence of agent B, might lead to different conclusions.

In conclusion, in this study we sought to investigate if anxiety states, induced via threat-of-shock, affect how social interactions are perceived. Namely, we meant to evaluate if our ability to extrapolate and apply expectations from communicative gestures to infer the presence of a second agent partaking in the interaction remains intact under anxiety. We saw no evidence that being under anxiety, compared to a safe/neutral contexts, affects our weighing of expectations in the perception of social scenes. This conclusion was further extended by the lack of association between anxiety questionnaires (state, trait and social) and decision criterion. Lastly, gaze analysis revealed that time spent collecting cues was similar across threat and safe contexts. Only some hints of a more erratic fixation pattern (shorter fixation times) were shown during threat in comparison to safe contexts. Thus, we found no evidence that being under a state of induced anxiety affects how expectations are formulated and used to anticipate social interactions.

## Figures and Tables

**Figure 5:**
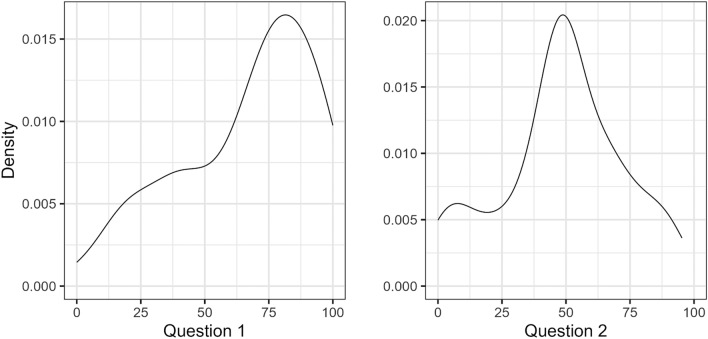
Density distributions of all responses in the last two questions.

**Figure 6:**
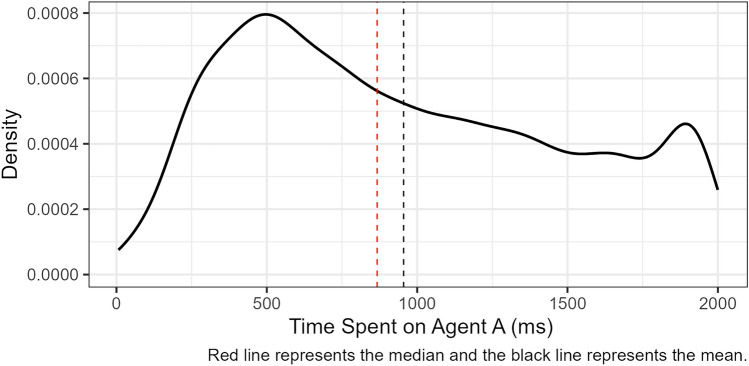
Distribution of time spent gazing at agent A during the first two seconds. Data represents all trials and all participants. The red line represents the median and the black line represents the mean.

## Data Availability

All data and analyses scripts are available at: https://osf.io/6vawb. All data and analyses scripts can be found in the same link.

## Appendix A: End questions

A graphical exploration (Fig. 5) of the questions asked at the end of the experimental task regarding (1) to what extent the participant used cues from agent A to infer the presence of agent B (0—“Not at all” to 100—“A lot”) and (2) how much the participant thought agent A and B’s actions were related (0—“Not at all” to 100—“A lot”), is shown below.

## Appendix B: Bayes factor analysis over criterion and sensitivity

We conducted two Bayesian analyses over our criterion and sensitivity measures, respectively. We used the brms package^61^ in R. For each, block type and action type were used as predictors. We chose non-informative priors for intercepts (Cauchy distribution with x_0_ = 0 and γ = 0.2) and betas (Cauchy distribution with x_0_ = 0 and γ = 0.5), with a sensitivity analysis supporting the robustness of this prior choice. We used 2000 iterations with a burn-in period of 1000 iterations. Convergence of the chains was assessed visually, through a trace plot inspection, and by calculating the Gelman-Rubin statistic. Bayes factors were computed using the bayestestR package for R^62^. Below the estimates and other statistics from both models (criterion and sensitivity) alongside the bayes factors are presented (Tables 2 and 3).

**Table 2:** Bayesian analysis for the criterion data. Each line represents each parameter of the final model.

Parameter	Est	Est. Error	l-95% CI	u-95% CI	Rhat	BulkESS	TailESS	BF_01_
Intercept	0.17	0.07	0.012	0.31	1.00	2935	3208	0.97
Block	0.03	0.10	− 0.18	0.22	1.00	2686	2799	6.25
Action Type	0.31	0.10	0.11	0.51	1.00	2652	2727	0.08
Block * Action Type	-0.03	0.14	− 0.31	0.24	1.00	2345	2541	4.9

**Table 3:** Bayesian analysis for the sensitivity data. Each line represents each parameter of the final model.

Parameter	Est	Est. Error	l-95% CI	u-95% CI	Rhat	BulkESS	TailESS	BF_01_
Intercept	0.53	0.1	0.33	0.73	1.00	3171	3013	0.001
Block	0.04	0.14	− 0.22	0.31	1.00	2797	2709	4.425
Action Type	0.11	0.14	− 0.16	0.4	1.00	2839	2927	3.413
Block * Action Type	0	0.18	− 0.38	0.36	1.00	2434	2628	3.718

## Appendix C: Time spent on agent A

The image below (Fig. 6) represents the distribution of time spent looking at agent A during the first two seconds of every trial across participants.
